# Commissioning and verification of the collapsed cone convolution superposition algorithm for SBRT delivery using flattening filter‐free beams

**DOI:** 10.1120/jacmp.v15i2.4631

**Published:** 2014-03-06

**Authors:** Ryan D. Foster, Michael P. Speiser, Timothy D. Solberg

**Affiliations:** ^1^ Department of Radiation Oncology University of Texas Southwestern Medical Center Dallas TX USA; ^2^ Department of Radiation Oncology Englewood Hospital and Medical Center Englewood NJ USA; ^3^ Department of Radiation Oncology University of Pennsylvania, Perelman Center for Advanced Medicine Philadelphia PA USA

**Keywords:** commissioning, beam modeling, flattening filter‐free, convolution superposition

## Abstract

Linacs equipped with flattening filter‐free (FFF) megavoltage photon beams are now commercially available. However, the commissioning of FFF beams poses challenges that are not shared with traditional flattened megavoltage X‐ray beams. The planning system must model a beam that is peaked in the center and has an energy spectrum that is softer than the flattened beam. Removing the flattening filter also increases the maximum possible dose rates from 600 MU/min up to 2400 MU/min in some cases; this increase in dose rate affects the recombination correction factor, $P_{\text{ion}}$, used during absolute dose calibration with ionization chambers. We present the first‐reported experience of commissioning, verification, and clinical use of the collapsed cone convolution superposition (CCCS) dose calculation algorithm for commercially available flattening filter‐free beams. Our commissioning data are compared to previously reported measurements and Monte Carlo studies of FFF beams. Commissioning was verified by making point‐dose measurement of test plans, irradiating the RPC lung phantom, and performing patient‐specific QA. The average point‐dose difference between calculations and measurements of all test plans and all patient specific QA measurements is 0.80%, and the RPC phantom absolute dose differences for the two thermoluminescent dosimeters (TLDs) in the phantom planning target volume (PTV) were 1% and 2%, respectively. One hundred percent (100%) of points in the RPC phantom films passed the RPC gamma criteria of 5% and 5 mm. Our results show that the CCCS algorithm can accurately model FFF beams and calculate SBRT dose distributions using those beams.

PACS number: 87.55.kh

## I. INTRODUCTION

Recently, medical linear accelerator manufacturers have begun offering linacs equipped with flattening filter‐free (FFF) beams. The Varian TrueBeam (Varian Medical Systems, Palo Alto, CA) and the Elekta Versa HD (Elekta AB, Stockholm, Sweden) offer high‐dose rate photon modes that are made possible by removing the flattening filter, which is normally in place to produce a uniform flat profile across the beam. Removing the filter results in a highly forward‐peaked beam profile, increases the dose rate, and softens the beam. While several recent papers have summarized the dosimetric characteristics of these beams^1^, ^2^, ^3^ and others have reported commissioning results of the anisotropic analytical algorithm^(4)^ or Monte Carlo,^5^, ^6^, ^7^ scarce data exist on modeling these beams with the collapsed cone convolution superposition (CCCS) algorithm as implemented in the widely used Pinnacle^3^ treatment planning system (Philips Radiation Oncology Systems, Fitchburg, WI). Stathakis et al.^(8)^ reported their experience commissioning the CCCS algorithm for 6 and 18 MV unflattened beams by overriding interlocks related to the flattening filter, but no patients were treated with this configuration. Huang et al.^(9)^ recently published results from an equivalent quality unflattened beam obtained by removing the flattening filter and tuning the electron energy in a Siemens Oncor linac (Siemens Medical Solutions, Concord, CA). They modeled the beam in Pinnacle^3^ and treated patients using this beam, which was matched in quality to a flattened 6 MV beam by tuning the electron energy until the $\%\text{dd}({10)}_{x}$ of the unflattened beam matched the flattened beam. In contrast, the commercially available FFF beams have a thin brass foil in place of the flattening filter, as opposed to the 1 mm steel plate in the Varian 23EX used by Stathakis, which will cause differences in the photon spectra and the beam model. By tuning the electron energy to achieve an equivalent quality unflattened beam as reported by Huang et al., many of the energy spectrum‐related model parameters would be similar to those of a flattened 6 MV beam. In Huang's study, the same energy spectrum was used to model the unflattened and the flattened beam. The majority of institutions commissioning FFF beams will be using commercially available solutions, rather than modifying linacs in‐house to produce FFF beams, and therefore the beam model parameters will be different than those for the flattened beams. Stathakis and Huang provide few details of the model parameters of their beams for the CCCS algorithm, a gap that this paper tries to fill. We report on the model parameters determined for 6 and 10 MV flattening filter‐free (FFF) beams for the CCCS algorithm and compare those parameters to the flattened beams. We also provide results of commissioning measurements and patient‐specific QA results as validation of the accuracy of our model.

## II. MATERIALS AND METHODS

A composite set of beam data from two Varian TrueBeam linear accelerators equipped with 6 and 10 MV FFF modes and the Millenium 120 leaf MLC was collected. Depth doses and off‐axis profiles were measured in a Wellhofer water phantom (IBA Dosimetry, GmbH, Germany) using IBA CC13 (0.13 cm^3^ volume and 6 mm diameter) and PTW 31014 (0.015 cm^3^ volume and 2 mm diameter; PTW, Freiburg, Germany) ion chambers and the Sun Nuclear Edge Detector SFD‐3G (0.0019 mm^3^ active detection volume and $0.8\times 0.8\;{\text{mm}}^{2}$ active detection area; Sun Nuclear Corporation, Melbourne, FL). The CC13 was used to scan field sizes $\geq 4\times 4\;{\text{cm}}^{2}$ and the Edge Detector was used to scan the smaller fields. We oriented the PTW 31014 vertically and scanned small fields to verify the Edge Detector scans, since it was a new detector to our clinic. All measurements were made with a source‐to‐surface distance of 100 cm SSD. Following the Pinnacle^3^ Beam Data Collection Guide, measurements were made for fields shaped by the jaws with the MLC fully parked and for fields shaped with the MLC and the jaws set at $20\times 20\;{\text{cm}}^{2}$. However, all data and results presented here were measured with the field defined by the jaws because the range of measured field sizes is greater. Crossline and inline profiles were measured at four depths ($d_{\max}$, 5, 10, and 20 cm) for each field size. Measured field sizes ranged from 1×1 up to $40\times 40\;{\text{cm}}^{2}$. All scans were postprocessed according to the guidelines provided in AAPM Task Group 106.^(10)^ Output factors were measured at a depth of 10 cm in water using the CC13 ion chamber for field sizes $3\times 3\;{\text{cm}}^{2}$ and larger, and the Sun Nuclear Edge Detector for the 1×1 and $2\times 2\;{\text{cm}}^{2}$ field sizes.

Reference dosimetry was performed according to AAPM's Task Group 51.^(11)^ During absolute calibration, we measured the recombination correction factor, $P_{\text{ion}}$ for the FFF beams. $P_{\text{ion}}$ depends on dose per pulse and will change if either the dose per pulse for a fixed dose rate or the dose rate changes.^(11)^ Without a flattening filter, the dose rates for the 6 MV FFF and 10 MV FFF beams are approximately 2.3 and 4 times higher than their flattened counterparts. Measurements of $P_{\text{ion}}$ for the FFF beams were compared to those for the flattened beams.

The beams were modeled using Pinnacle^3^ version 9.0 (Philips Medical, Milpitas, CA). Pinnacle^3^'s automodeling library was used to tune the model to match the measured profiles. Profiles were then manually adjusted to obtain the best fit to the measurements. Profile calculations were performed with a phantom dose grid size of 0.1 cm. Agreement of the model with measured data was evaluated using Pinnacle^3^'s built‐in tools which compute the percent difference and distance‐to‐agreement between the curves. The percent error for the percent depth dose curves is calculated using Eq. (1) and for the off‐axis profiles using Eq. (2):

(1) $$\%\;\text{Error}=(\text{model}‐\text{measured})/D_{\text{max}}$$

(2) % Error=(model‐measured)/central axis dose

The depth dose curves are analyzed in three sections according to depth. The off‐axis profiles are separated into “inner beam” and “outer beam” and these regions are defined as in AAPM Task Group 53.^(12)^ The inner beam is defined as the high‐dose area 0.5 cm inside the geometric field edge and the outer beam is the low‐dose region 0.5 cm outside the field edge. The data for the off‐axis profiles in the tables are for the crossline scan at a depth of $d_{\text{max}}$. Beam model parameters of the FFF beams were compared with those of the flattened 6 and 10 MV beams. The penumbra for the FFF beams was calculated using the method detailed by Ponisch et al.^(13)^ A detailed description of the CCCS beam model parameters and their effects on the model has been given previously^(14)^ and will not be covered here.

Commissioning was verified by planning several test cases on existing patient CT scans and delivering these plans to phantoms. Because the intent was to use the FFF beams for high dose per fraction clinical delivery, test plans consisted of fixed beam conformal lung and dynamic conformal arc techniques for lung SBRT and IMRT for prostate SBRT. Doses ranged from 10 to 18 Gy per institutional clinical protocols. To verify the accuracy of the commissioning, point doses were measured using a PTW 31014 ion chamber and planar dose distributions were mea‐sured using GAFCHROMIC EBT3 (ISP Corporation, Wayne, NJ) film for the SBRT prostate plan, which was measured in a homogenous solid water phantom $\left(30\times 30\times 22\;{\text{cm}}^{3}\right)$, while the lung cases were measured in a specially designed anthropomorphic thorax phantom (Integrated Medical Technologies, Troy, NY). The thorax phantom contains unit density targets imbedded in lung to allow direct verification of dosimetric calculations in heterogeneous media, but does not allow for planar film measurements in the vicinity of the lung targets. Additionally, prior to treating patients, the Radiological Physics Center's (RPC) lung phantom was irradiated as an independent check of the commissioning.^(15)^ The National Cancer Institute (NCI) requires institutions wishing to participate in NCI‐sponsored clinical trials to image the phantom, create a plan to deliver 6 Gy to the PTV, perform their customary patient specific QA for the phantom plan, and irradiate the phantom. The RPC reads the dose as measured by TLD capsules inside the phantom and analyzes films to determine the dose distributions in axial, coronal, and sagittal planes inside the phantom. Measured doses and distributions determined by the RPC are compared to reported values from the institution's planning system. Patient‐specific QA was performed for the two patients (four tumors) treated to date with FFF beams, and those results are also reported here.

## III. RESULTS

**Figure 1:**
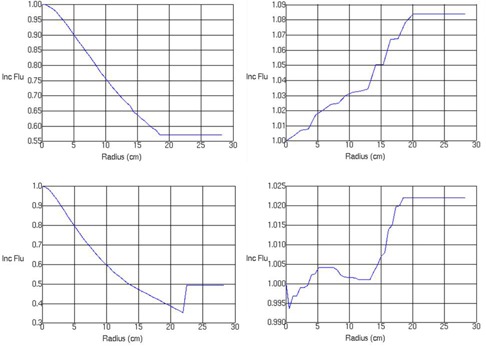
Arbitrary fluence profiles for 6 MV FFF (top left), 6 MV (top right), 10 MV FFF (bottom left), and 10 MV (bottom right).

**Figure 2:**
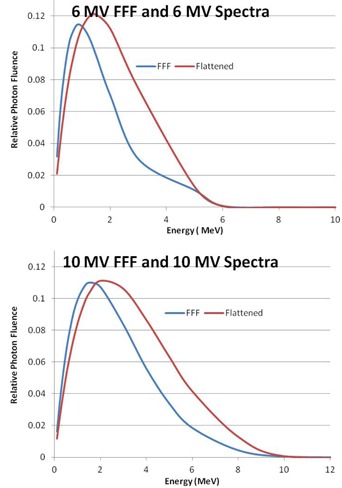
Photon spectra for 6 MV FFF and 6 MV (top), and 10 MV FFF and 10 MV (bottom).

The beam model parameters are found in Table 1. The arbitrary fluence profiles for 6 MV FFF, 6 MV, 10 MV FFF, and 10 MV beams are shown in Fig. 1. These profiles describe the incident photon fluence as a function of distance from the central axis, which is dependent on the flattening filter attenuation. The FFF profiles are substantially different from their flattened counterparts, exhibiting a decrease in fluence as a function of radius from the central axis as opposed to an increase. The photon energy spectra for 6 MV FFF and 10 MV FFF are shown in Fig. 2, along with the flattened 6 and 10 MV spectra. The difference in beam quality between the flattened and FFF beams is readily apparent from the plotted spectra; the FFF beams are shifted towards lower energy bins. The beam modeling optimizer was able to obtain very good agreement with the measured profiles, as shown in 3, 4. Depth dose curves and profiles shown are for 1×1,10×10, and $20\times 20\;{\text{cm}}^{2}$ field sizes. A single model was generated for all field sizes; we did not find it necessary to split the model for different field sizes in order to get good agreement with measurements. 2, 3 contain the maximum percent error between the measured data and the model as reported by Pinnacle^3^. Penumbra at a depth of 10 cm and the distance to agreement (DTA) for the off‐axis profiles are also reported.

**Table 1:** Pinnacle^3^ beam model parameters for 6 and 10 MV FFF compared to flattened 6 and 10 MV beams. For explanation of these parameters, see Starkschall et al.^(14)^

*Parameter*	*6 MV FFF*	*6 MV Flattened*	*10 MV FFF*	*10 MV Flattened*
Source A‐B dimension (cm)	0.0562	0.05	0.1112	0.0575
Source G‐T dimension (cm)	0.0787	0.05	0.0778	0.0974
Gaussian height (cm)	0.03324	0.06	0.02838	0.07814
Gaussian width (cm)	1.359	1.2	1.332	1.3647
Off‐axis softening factor	0.84375	7	3.1875	14.2234
X collimator transmission (%)	0.00626	0.00149	0.00339	0.00149
Y collimator transmission (%)	0.00626	0.00149	0.00339	0.00149
MLC transmission (%)	0.0133	0.015	0.015	0.020
Leaf tip radius (cm)	8	8	8	8
Tongue‐and‐groove width (cm)	0	0	0	0
Interleaf leakage transmission (%)	0	0	0	0
Max depth (cm)	3.3	3	3.3	3.3
EC surf dose	0.7130	0.5878	0.2937	0.2040
Depth coefficient	9.9014	3.4248	4.0115	1.8109
Off‐axis coef	0	0	0	0
DF	0.008475	0.008762	0.0005346	0.0008603
SF	0.916	0.950	0.779	0.974
C1	0.0177	0.00528	0.00269	0.00405
C2	−1.220	0.245	0.207	0.471
C3	0.00847	0.1923	0.0964	0.0789

The output factors are plotted in Fig. 5. Output factors for FFF beams are larger than those for the flattened beams for field sizes below $10\times 10\;{\text{cm}}^{2}$ and smaller than the flattened beam output factors for fields larger than $10\times 10\;{\text{cm}}^{2}$. This pattern persists when we calculate output factors at $d_{\text{max}}$ and is also reported by other authors for the TrueBeam^1^, ^2^, ^4^ and Varian 21EX.^(16)^ Our measured output factors agree better than 1% with those reported by Gete et al.,^(7)^ except for the 1×1 field which is 1% different from their Monte Carlo calculation.

Pinnacle^3^ calculates an output correction factor, ${\text{OF}}_{c}$, which corrects for collimator scatter not already included in the beam model. This factor is used to correct the incident energy fluence and should be uniform among the range of field sizes, per the Pinnacle^3^ Physics Reference Guide. If all the collimator scatter effects have been incorporated into the model, ${\text{OF}}_{c}$ should be 1.000 for all field sizes; thus, ${\text{OF}}_{c}$ is an indication of the quality of the beam model. For field sizes ranging from 1×1 to $40\times 40\;{\text{cm}}^{2}$, the maximum difference in any two field size's ${\text{OF}}_{c}$ values was 4.1% for 6 MV FFF and 2.4% for 10 MV FFF. The largest deviation from unity for either energy was the 6 MV FFF $1\times 1\;{\text{cm}}^{2}\;{\text{OF}}_{c}$ of 0.968. ${\text{OF}}_{c}$ values are reported in 2, 3.

**Figure 3:**
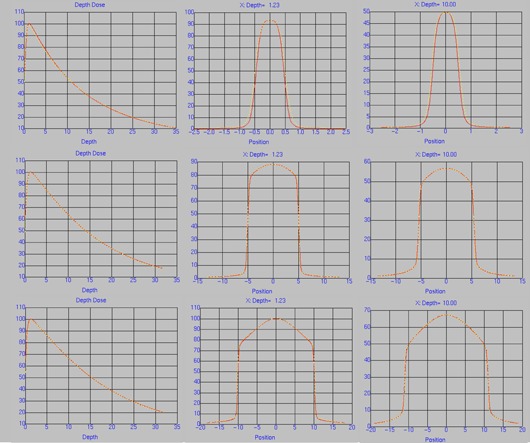
Measured (red) and modeled (yellow) PDD and profiles at depth 1.23 cm and 10.0 cm for 6 MV FFF for 1×1 (top), 10×10 (middle), and 20×20 (bottom) cm^2^ field sizes.

$P_{\text{ion}}$ for 6 MV FFF was measured to be 1.0046 at a dose rate of 1400 MU/min and 1.001 at a dose rate of 600 MU/min for the 6 MV flattened beam. For the 10 MV FFF beam, $P_{\text{ion}}$ was 1.008 at a dose rate of 2400 MU/min and 1.0026 with a dose rate of 600 MU/min. A decrease in ion collection efficiency was observed due to the higher dose rates of the FFF beams, which has also been observed in other reports.^1^, ^2^, ^17^

The point dose and film analysis (where applicable) results from the commissioning verification measurements and the patient‐specific QA measurements are presented in Table 4. We have treated two patients with FFF beams thus far. The first patient had three separate tumors, each treated with its own isocenter. Including the test plan measurements and the QA performed on the RPC phantom's plan, eight point dose measurements are presented. Our institutional gamma criteria for film analysis of planar dose distributions for IMRT plans are 3% and 3 mm. For the prostate SBRT plan, 95.9% of pixels in the high‐dose region passed the gamma analysis, as shown in Fig. 6. The anthropomorphic thorax phantom used for the patient QA and verification plans is shown in Fig. 7. Compared to four lung SBRT patients treated in our clinic on the TrueBeam with flattened beams (data not shown), the plans using FFF beams had slightly fewer MU per prescribed Gy and an average QA point dose difference that was less than half of that for the flattened beam QAs.

**Figure 4:**
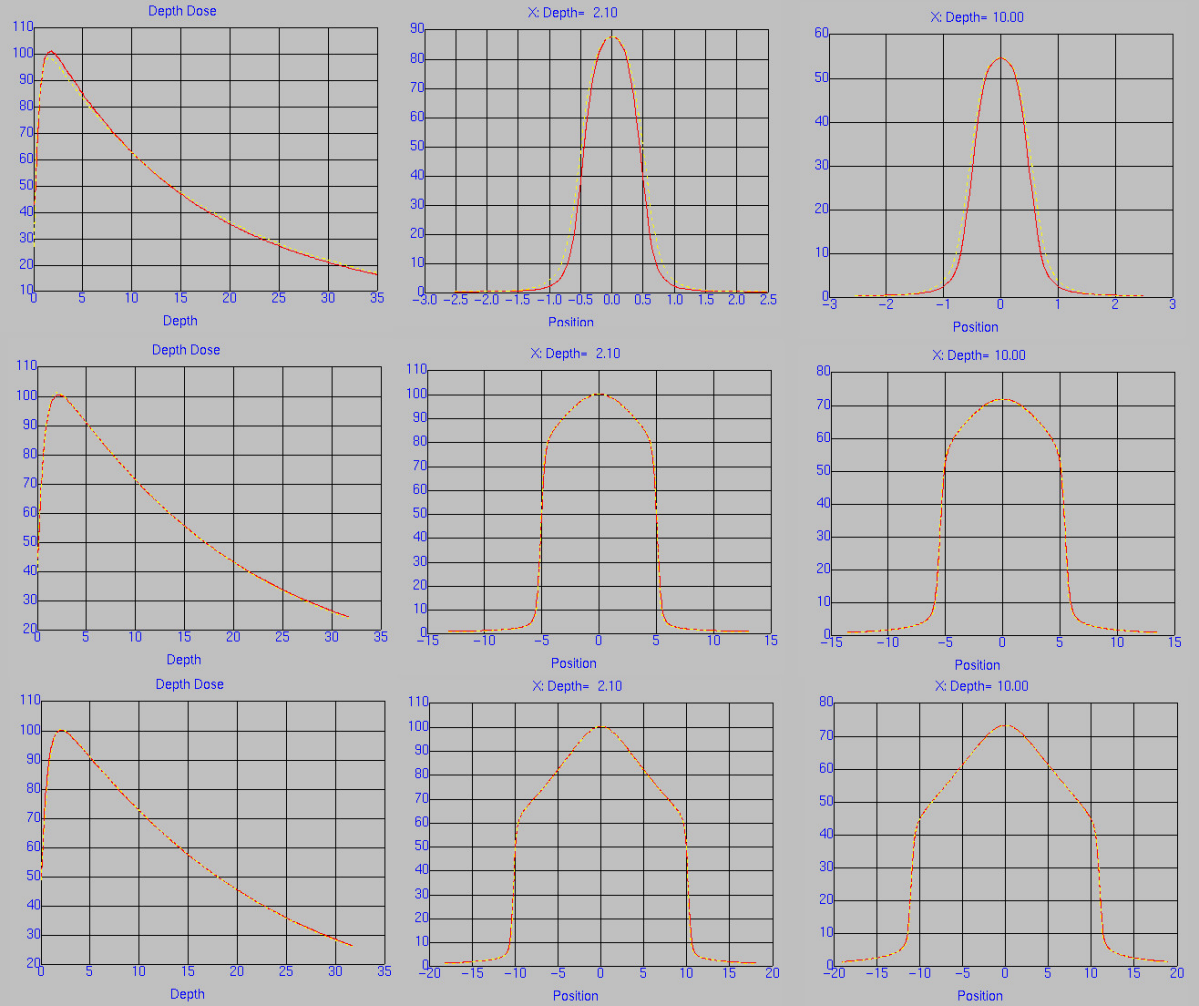
Measured (red) and modeled (yellow) PDD and profiles at depth 2.10 cm and 10.0 cm for 10 MV FFF for 1×1 (top), 10×10 (middle), and 20×20 (bottom) cm^2^ field sizes.

**Table 2:** Maximum percent error between measured and modeled profiles

*6 MV FFF*									
*Field Size*	$d_{max}(cm)$	$1\;cm-d_{max}$	$d_{max}-19\;cm$	19−30 cm	*Off‐axis Inside Beam*	*Off‐axis Outside Beam*	*Penumbra (mm)*	*FHWM DTA (mm)*	$OF_{c}$
1×1	1.20	1.55%	2.12%	0.39%	0.15%	1.12%	2.4	0.5	0.968
2×2	1.30	0.27%	1.24%	0.09%	0.34%	0.85%	2.8	0.4	0.980
5×5	1.32	0.44%	0.72%	0.37%	2.39%	0.83%	5.6	0.2	0.992
10×10	1.30	0.50%	0.26%	0.30%	1.70%	0.78%	6.6	0.5	1.000
15×15	1.32	0.70%	0.48%	0.24%	1.52%	1.02%	7.4	0.2	1.000
20×20	1.27	0.89%	0.33%	0.23%	1.30%	0.80%	7.9	0.8	1.005
30×30	1.22	0.73%	0.32%	0.20%	0.61%	1.32%	9.2	1	1.008
40×40	1.28	0.49%	0.34%	0.16%	1.71%	1.96%	10.0	1.2	1.005

The RPC lung phantom results are shown in Table 5. Our institution has irradiated the RPC lung phantom two other times with different planning system‐linac combinations and, while all three irradiations met the RPC criteria, the irradiation with the FFF beams was superior to the other two. Ratios of the PTV TLD doses for the other irradiations ranged from 0.95 to 0.98, and the percentage of points passing the gamma index ranged from 96% to 97%. Considering the first time pass rate of the RPC lung phantom is only 71%,^(18)^ it represents a rigorous test for the FFF beam modeling and commissioning.

**Table 3:** Maximum percent error between measured and modeled profiles

*10 MV FFF*									
*Field Size*	$d_{max}(cm)$	$1\;cm-d_{max}$	$d_{max}-19\;cm$	19−30 cm	*Off‐axis Inside Beam*	*Off‐axis Outside Beam*	*Penumbra (mm)*	*FHWM DTA (mm)*	$OF_{c}$
1×1	1.80	2.98%	3.07%	1.11%	0.20%	1.88%	3.1	0.5	0.985
2×2	2.27	2.54%	0.71%	0.48%	0.23%	2.00%	3.6	0.4	0.988
5×5	2.18	2.02%	0.68%	0.59%	2.15%	1.43%	6.6	0.4	0.997
10×10	2.13	1.32%	0.46%	0.58%	1.16%	1.81%	6.8	0.4	1.000
15×15	2.07	1.04%	0.30%	0.43%	0.82%	1.86%	7.1	0.4	1.000
20×20	2.08	2.11%	0.46%	0.43%	0.41%	1.44%	7.6	0.5	1.006
30×30	2.07	2.94%	0.33%	0.28%	0.87%	0.82%	8.5	0.5	1.009
40×40	2.11	2.99%	0.42%	0.17%	0.995	1.25%	9.1	0.8	1.009

**Figure 5:**
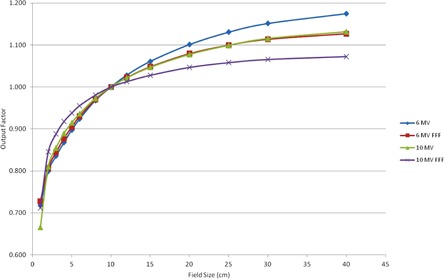
Plot of output factors measured at 10 cm depth and 100 cm SSD.

**Table 4:** Commissioning model verification measurements and patient‐specific QA results

*Site/Plan*	*Energy*	*Beams*	*Dose/fx (Gy)*	*Point Dose % Difference*	*% Pixels Passing* ^a^
Lung SBRT ‐ 3D CRT	6 FFF	10	18	0.82%	N/A
Lung Conformal Arc	6 FFF	4	18	−0.22%	N/A
SBRT Prostate	10 FFF	13	10	−1.15%	95.90%
Patient 1 Tumor 1 Lung SBRT ‐ 3D CRT	6,10 FFF	10	10	1.53%	N/A
Patient 1 Tumor 2 Lung SBRT ‐ 3D CRT	6,10 FFF	10	10	2.09%	N/A
Patient 1 Tumor 3 Lung SBRT ‐ 3D CRT	6,10 FFF	10	10	0.84%	N/A
Patient 2 Lung SBRT ‐ 3D CRT	6 FFF	10	11	1.37%	N/A
RPC Lung Phantom SBRT ‐ 3D CRT^b^	6 FFF	10	6	1.11%	N/A
			Average	0.80%	

^a^ 3%, 3 mm gamma criteria for high dose region.

^b^ Result of patient specific QA measurement of the phantom plan before phantom irradiation.

**Figure 6:**
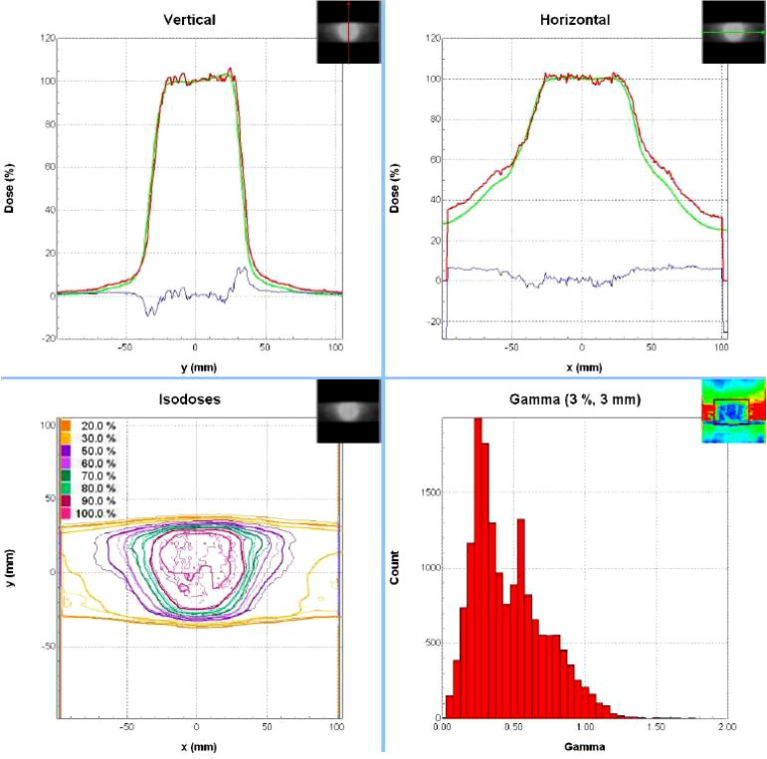
The film analysis result for the SBRT prostate IMRT plan.

**Figure 7:**
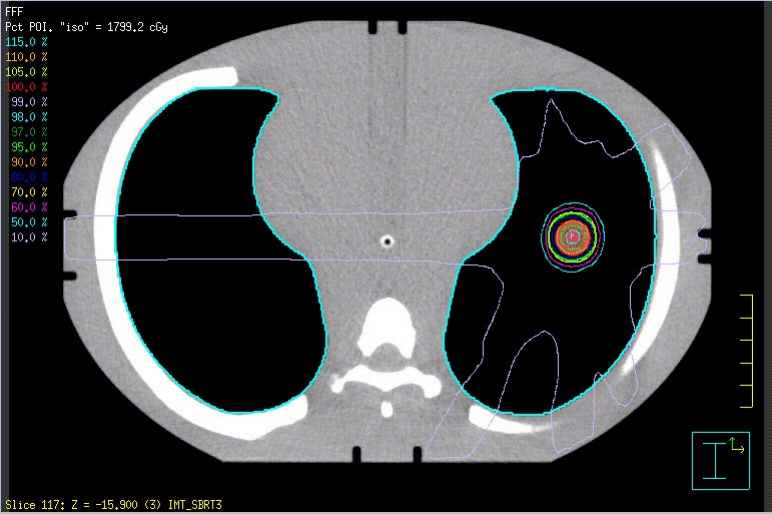
The anthropomorphic thorax phantom used for patient‐specific lung SBRT QA.

**Table 5:** Summary of RPC lung phantom results.

*Location*	*RPC vs. Institution*	*Criteria*
PTVTLDsup	0.98	0.92−1.02
PTVTLDinf	0.99	0.92−1.02
*Film Plan*	*Gamma Index* ^a^	*Criteria*
Axial	100%	≥80%
Coronal	100%	≥80%
Sagittal	100%	≥80%
Average over 3 planes	100%	≥85%

^a^ Percentage of points meeting gamma‐index criteria of 5% and 5 mm.

## IV. DISCUSSION & CONCLUSIONS

We have demonstrated that the CCCS algorithm can accurately model commercially available flattening filter‐free megavoltage photon beams. As has been described previously, we found that some model parameters are not necessarily representative of the physics it attempts to model.^(19)^ For example, our beam modeling determined the maximum depth for electron contamination in the 10 MV flattened beam to be the same as the 6 MV FFF beam (see the Max Depth parameter in Table 1). This is an unexpected result because the 6 MV FFF beam is a softer beam and the maximum depth of contamination electrons in a 6 MV FFF beam should be shallower. However, these model parameters provide an accurate dose calculation when compared to measurements. For the majority of the differences in 2, 3, the agreement between the model and the measurements meets the recommended criteria from Task Group 53. Output factors and $P_{\text{ion}}$ values agreed well with other published results from TrueBeam linacs. Verification plans and patient‐specific QA measurements demonstrate excellent agreement with calculations, with an average difference between calculation and measurement of less than 1%. The RPC lung phantom results were particularly good, with absolute dose differences ≤2% and 100% of points passing gamma index criteria of 5% and 5 mm under heterogeneous conditions. The excellent agreement between calculation and measurement indicates that the planning system is able to accurately model and calculate dose for FFF beams, even for complex plans delivered to heterogeneous phantoms. While it's not expected that every TrueBeam with flattening filter‐free beams will have identical model parameters, we believe this report provides a good starting point for modeling these beams and will be a reference for future commissioning efforts at other institutions.

## ACKNOWLEDGMENTS

We would like to thank Catherine Large at Philips Radiation Oncology Systems for her helpful suggestions during the beam modeling and commissioning process.

## Supporting information

Supplementary Material

Supplementary Material
