# Supplementary figures and images for: Commissioning and verification of the collapsed cone convolution superposition algorithm for SBRT delivery using flattening filter‐free beams
Source: J Appl Clin Med Phys. 2014 Mar 6;15(2):39–49. doi: 10.1120/jacmp.v15i2.4631 (PMC5875462; doi:10.1120/jacmp.v15i2.4631)

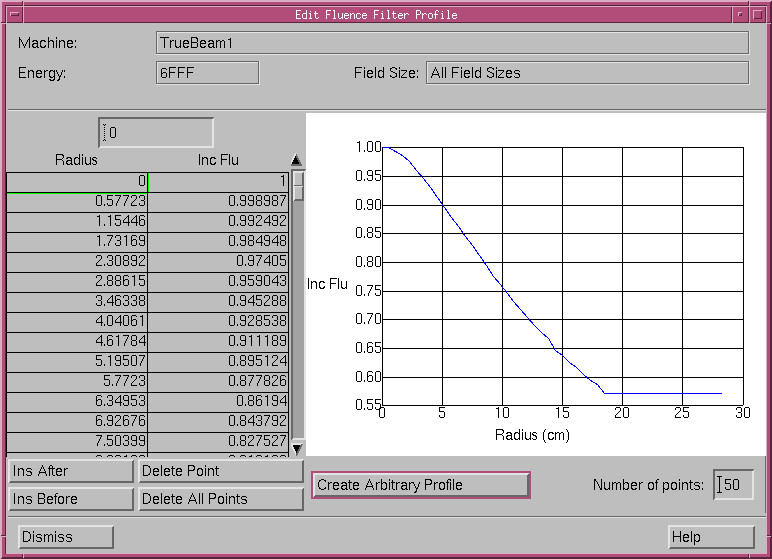

Supplement: Supplementary file 1 — Supplementary Material [file ACM2-15-39-s001.jpg]

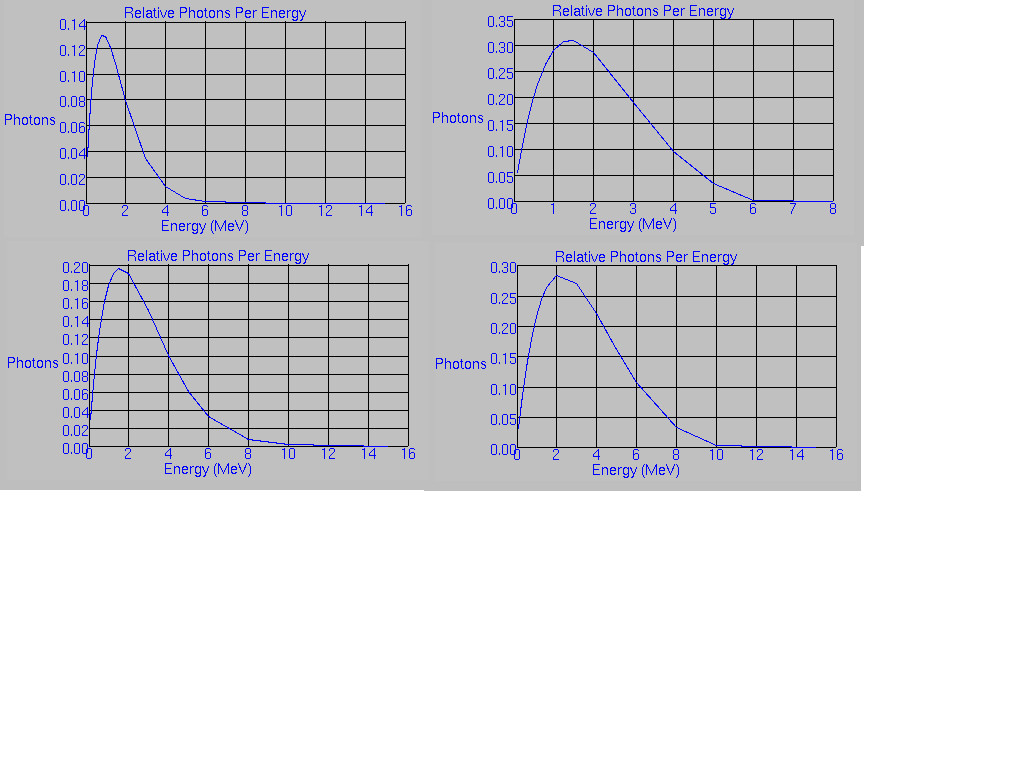

Supplement: Supplementary file 2 — Supplementary Material [file ACM2-15-39-s002.jpg]
